# Challenges during the realization of an international research project on leishmaniasis in Colombia

**DOI:** 10.3389/fpubh.2023.1143939

**Published:** 2023-04-04

**Authors:** Raluca Suschinel, Aylen Lisset Jaimes-Mogollón, Reinaldo Gutiérrez-Marín, Luís Carlos Peña-Cortés, Jesús Alberto Mendoza-Ibarra, José Flórez-Gélvez, Cătălin Dumbravă, Marius Uzunof, Violeta Elena Simion, Radu Ionescu

**Affiliations:** ^1^Institute of Veterinary Medicine and Animal Sciences, Estonian University of Life Sciences, Tartu, Estonia; ^2^GISM Group, Faculty of Engineering and Architecture, University of Pamplona, Ciudad Universitaria, Pamplona, Colombia; ^3^GIEPATI Group, Faculty of Health, University of Pamplona, Ciudad Universitaria, Pamplona, Colombia; ^4^GICA Group, Faculty of Agricultural Sciences, University of Pamplona, Ciudad Universitaria, Pamplona, Colombia; ^5^Faculty of Veterinary Medicine, Spiru Haret University, Bucharest, Romania

**Keywords:** public health, international cooperation, Colombia, leishmaniasis, dogs

## Abstract

Leishmaniasis is an infectious disease that belongs to the top 10 neglected tropical diseases. It mainly affects the poor population from tropical and subtropical areas of the World, which lacks sufficient resources and means to fight against this disease. With this in mind, the European Commission has funded an international collaborative research project in which are participating various institutions from South America, North Africa and Europe. The main objective of this project is the development of a fast, less expensive, non-invasive and easy to use alternative method for leishmaniasis diagnosis in dogs, one of the main reservoirs of leishmaniasis spread to humans. In this perspective article, we present our personal insight and opinion regarding the challenges of realizing a joint international research project on leishmaniasis in Colombia, a country where leishmaniasis is endemic, as well as regarding the involvement of the Public Health institutions and the local population from this country.

## 1. Introduction

Leishmaniasis is a parasitic disease produced by the protozoa of the genus Leishmania that affects both animals and humans. It is transmitted through the bite of female sandflies, which ingest the parasite during the process of feeding with blood from a person or animal infected with the parasite, transmitting it afterwards to an uninfected animal or human being (1).

At the global level, leishmaniasis is spread mainly in the tropical and subtropical areas of the World, where the habitat of the insects that transmit the disease is mainly found. These areas include Central and South America, northern and eastern parts of Africa, eastern and southeastern Asia, the Middle East, and the Mediterranean basin area in southern Europe (2).

The global prevalence of leishmaniasis in humans is estimated to be of approximately 12 million cases, with an annual incidence rate of 1.3 million new cases and an annual death rate of approximately 70,000 people (3). Depending on the form of the disease, it is worth noting that approximately 80% of the cases of cutaneous leishmaniasis (one of the two main forms of the disease, characterized by ulcerations produced on the skin) have been reported in Colombia, Brazil, Iraq, Pakistan, Syria, Afghanistan and Algeria (4), whereas 90% of the cases of visceral leishmaniasis (the other main form of the disease, which affects the spleen, liver and bone marrow) have been reported in Brazil, Ethiopia, India, Bangladesh, Sudan and South Sudan (5).

The disease mainly affects the poor population, whereas its prevalence is higher in the rural and peri-urban areas and in jungle environment (6). Malnutrition, population displacement, housing with poor living conditions, lack of resources and weak immune system are other important factors that favor the transmission of this disease (7). Leishmaniasis is also linked to environmental changes such as deforestation, urbanization, construction of dams and irrigation systems (8).

Thus, the most exposed people to this disease belong to the poorest strata of the population, with inadequate housing conditions and low socio-economic position in the society, for which the access to treatment can be furthermore prohibitive, besides of being challenging (9). If left untreated, the cutaneous form of leishmaniasis can cause permanent signs such as deformity and disfigurement, while the visceral form can cause death in more than 90% of the untreated cases (10).

Early and accurate diagnosis of this disease are essential for prescribing an adequate treatment and preventing the further transmission of the disease, as well as for improving the quality of life of the patients. Visual inspection of the patient is the first step in identifying certain signs compatible with Leishmaniasis (sores, weight loss, fever, enlargement of spleen and liver), but the clinical manifestations of the disease are not specific only to leishmaniasis, whereas some patients can have a silent infection without presenting symptoms or signs (11). Confirmatory tests, which include different parasitological, serological and molecular techniques (e.g., ELISA, indirect fluorescence antibody test, rapid immunochromatographic test, PCR), are expensive and time-consuming, and are not always available in routine daily practice in the disfavored areas where the disease is predominantly spread (12).

In light of these considerations, research is being performed for developing alternative diagnostic procedures for leishmaniasis, which should be less expensive, provide rapid results and could be performed on-site with minimal preparation. Such diagnostic procedures need to be extended also to animals, mainly dogs, which may be considered potential reservoirs for leishmaniasis transmission to humans (13), although their roles as reservoirs need to be adequately demonstrated (14, 15). Poverty is highly correlated with cohabitation with a high number of mongrel dogs, which is an important risk factor that further favors the spreads of the disease to the vulnerable population (16).

In this regard, we have performed various field activities in Colombia in the framework of an international research project that is aiming the development of a volatile test for non-invasive, easy and fast diagnosis of leishmaniasis in dogs, based on the analysis of their exhaled breath and of the volatiles released by their hair, an approach that was previously assessed to detect dogs with visceral leishmaniasis in Brazil (17). Our study is justified in the context of the epidemiologic control fragility of canine leishmaniasis in Colombia, which occupies the second place after Brazil in the number of cases of canine leishmaniasis in the Latin America region (15). The cases of human leishmaniasis are also seen as a health risk problem in Colombia, especially for the adult males, as they become infected when they enter the vector's biotopes to tap natural resources, or into the jungle for illicit crops culture, guerrilla-type activity or military actions to fight against the anterior ones, where the soldiers are accompanied by military dogs trained to detect landmines, which are exposed to sandflies infected with the leishmania parasite and are prone to acquire the disease (15, 18).

In this article we wish to present our viewpoint regarding the attitude and involvement of the Public Health institutions and dog owners from endemic zones of leishmaniasis in Colombia that we contacted for conducting our research, as well as the challenges that we needed to overcome.

## 2. Research project and activity performed

Research activities were performed in Colombia in the framework of the international research project CANLEISH funded by the European Commission (19), which is aiming the investigation and development of an alternative method for the diagnosis of leishmaniasis in dogs based on volatile samples analysis. The execution of this project was approved by the Committee for Ethics and Environmental Impact in Research of University of Pamplona, Colombia (Approval Certificate No. 002 from April 14th, 2021).

Various regions from Colombia affected by outbreaks of canine leishmaniasis, as well as isolated cases, were identified from the information published by the National Institute of Health from Colombia. Both cutaneous and visceral forms of canine leishmaniasis were considered, for better encompassing the different manifestations of this disease.

Leishmaniasis diagnosis at the dogs included in this study followed standard procedures. In the case of the dogs with skin lesions compatible with cutaneous leishmaniasis, smear samples were taken from the skin lesions for microscopic examination and parasite identification. Biopsies were also taken from the lesion in order to perform PCR analysis. In the case of the dogs suspected for visceral leishmaniasis, approximately 3 ml of blood samples were taken from the cranial area of the forepaw of the animal in order to apply the rapid immunochromatographic test for the rK39 antigen. Biopsies were moreover taken, employing the thin needle aspiration procedure, from the popliteal lymph nodes, located in the caudal area of the hind paw of the animal, when these nodes presented swelling effect. Before taking the biopsy, the animals were tranquilized using Tranquilan^®^ (Acepromazine) at a commercial dose (1 mg per kg of body weight, IV). From the sacrificed animals, spleen biopsies were taken after euthanasia by thin fine needle aspiration biopsy. Euthanasia was performed by administration of Eutanex^®^ (sodium pentobarbital 390 mg/ml, sodium diphenylhydantoin 50 mg/ml) at a commercial dose (1 ml per each 5 kg of body weight, IV). The biopsies were examined by PCR analysis.

**Figure 1:**
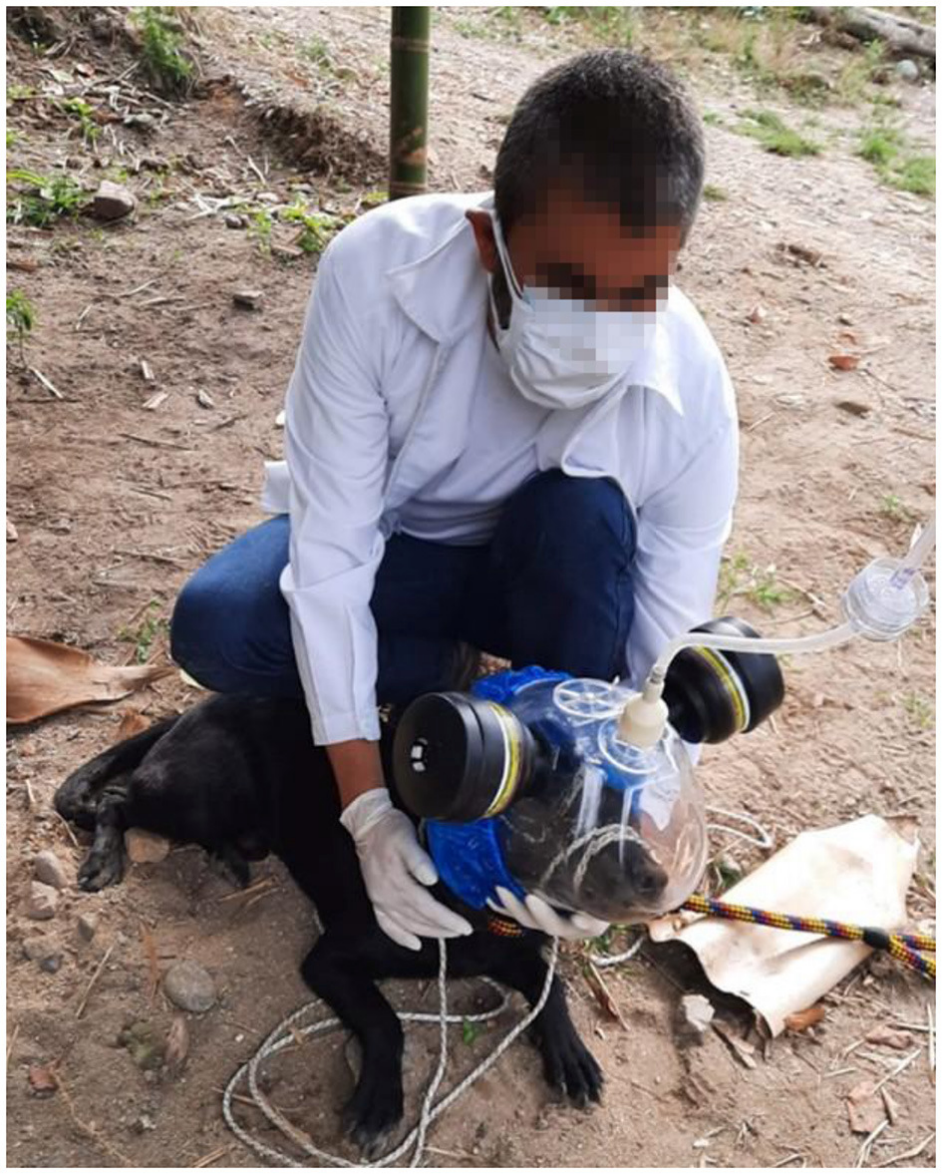
Mask used during breath sampling.

Specific field work related with the realization of the CANLEISH project consisted in the collection of different samples from the dogs for volatile analysis. A specific protocol was developed for this aim. Breath samples were taken by introducing dog's head inside an equine nebulization mask, which was adjusted such that to prevent the escape of animal's head (Figure 1). The volatile compounds emitted by dogs through breathing were acquired in Tenax sorbent tubes by pumping, as previously reported (20). Nervous dogs were tranquilized with Tranquilan^®^ at a commercial dose (1 mg per kg of body weight, IV) before performing this process. Additionally, approximately 10 mg of hair was cut near the lesion and stored in 125 ml wide mouth specimen jars. At a later stage, the volatiles released by hair were transferred to Tenax sorbent tubes through pumping, inside a biosafety cabinet for avoiding external contamination, in a similar way as reported for the acquisition of the volatiles emitted by feces (20). The volatiles stored in the Tenax tubes are currently analyzed employing various volatolomic techniques, and the results obtained will be published elsewhere.

## 3. Involved participants

### 3.1. Researchers

Following the specific rules of the project in whose framework the activities of this study were performed, which is based on the exchange of researchers between the participating institutions, researchers from three different countries took part at the field work. Researchers from Colombia, the country where the animals were selected and sampled, included veterinary, parasitology and pathology professionals, with experience in different diagnostic methods for infectious disease, and an electronic engineer. Researchers from veterinary institutions from two European countries, Estonia and Romania, have also participated. The European team included veterinary medicine students and a senior researcher with experience in sensor systems development for volatile samples analysis.

### 3.2. Public authority

The aim of this study was communicated to representatives of the regional Public Health Authorities and Mayor's Offices from the Departments^1^ of Colombia where the animals were selected for the study. The participation of the Public Authorities was in agreement with the operative procedures outlined by the Health and Social Protection Ministry of Colombia (21).

In the Department of Huila, where dogs with visceral leishmaniasis were sampled, representatives from the Departmental Health Secretary of Huila accompanied the researchers during their field work activities, and provided them assistance in detecting possible cases of canine leishmaniasis and dogs' sampling. The Public Authority staff that participated at the field work included three veterinarians, one biologist and 14 technicians from the Public Health Department, as well as six municipal employees.

In the Department of Norte de Santander, where dogs with cutaneous leishmaniasis were sampled, it was instead medical staff from the Public Health Services of various villages where animals were sampled who provided the same assistance to the researchers from the project.

### 3.3. Dog owners

Dog owners were inhabitants from rural and peri-urban areas with deficiencies in public services and a high level of poverty, lacking of basic sanitation or hygiene facilities. They were previously informed about the aim of the study and the day of dogs' sampling by the representatives of the local Public Health Authorities. Those who accepted their dogs to be included in the study received, in the day of sampling, detailed information from the researchers about the project, regarding both the objective of the study and the sampling procedure. They were also informed about researchers' obligation to communicate the results of the official standard tests for leishmaniasis realized in parallel with this study to the public health authorities in case of a positive result. The dogs were included in the study and sampled only after their owners signed the informed consent.

## 4. Discussion

### 4.1. Research framework

With the objective to provide useful means for community-based interventions aimed at the prevention and control of the spread of leishmaniasis, a disease that belongs to the top 10 neglected tropical diseases as from Pan American Health Organization (22), the European Commission funded an international collaborative research project in which different institutions from Colombia, Europe and North Africa are participating (19).

Although leishmaniasis is not generally present in most of the European countries, where its prevalence is mainly limited to the Mediterranean basin area, the adoption of dogs from southern Europe to other European countries led to the expansion of the number of reported cases in other European countries such as Great Britain or Germany (23). The European veterinarians are not however much exposed to canine leishmaniasis cases, therefore their ability to accurately diagnose or recognize the signs of this disease is very limited.

Actually, in accordance with European legislation, Directive 2003/99/EC of the European Parliament and of the Council amended by Council Directive 2013/20/EU, leishmaniasis is not included on the list of parasitic zoonoses that must be monitored within the epidemiological surveillance in Europe. Knowing the challenges of the situations when this disease occurs, the international collaboration within this project is helping researchers, authorities and the population from Europe to be better informed and to be able to manage this zoonosis and the problems faced by the public health also in Europe. In this regard, the research secondments performed in the framework of the CANLEISH project by staff from veterinary institutions from Estonia and Romania in Colombia, a country where leishmaniasis is endemic, represents an unvaluable opportunity for them for changing experiences in the diagnosis of this disease with their more experienced Colombian colleagues.

On the other hand, this project offered also a unique opportunity for the Colombian authorities, veterinarians and researchers to meet and collaborate with their counterparts from Europe during the realization of joint research and field activities in Colombia. The importance of this collaboration was reflected by the reception of the researchers in the Mayoralty of various towns from Colombia, and by the highlights published by local newspapers from Colombia regarding the project (24, 25).

In this perspective paper we do not however aim to present the scientific results of this study, which will be published elsewhere after the volatile samples collected from dogs during the field work performed in Colombia will be exhaustively investigated. Instead, we are focusing here in presenting our personal insight and opinion regarding the experiences and challenges of realizing a joint international research project on leishmaniasis in Colombia.

### 4.2. Public health authority involvement

Representatives of the public health authorities accompanied the researchers on the field, acting as an intermediary between the local population and the researchers. Their level of involvement and interest for the project were slightly different, depending on the spread of the disease and its effects in the specific territories where dogs were sampled.

In Colombia, human and canine visceral leishmaniasis are restricted to two major transmission foci, the Caribbean coast and middle Magdalena River Valley (26). The city of Neiva, capital of the Department of Huila, situated on the middle Magdalena River Valley, had suffered not long ago outbreaks of visceral leishmaniasis that produced the death of a baby child that acquired the disease from an infected dog (27). As a consequence, the Departmental Health Secretary of Huila started a very intensive campaign in the peri-urban area of Neiva aiming to control the disease and stop its further spread, to which it dedicates a team of 25 public servants and economical recourses of more than 45,000 USD / month for fighting against both leishmaniasis and dengue (another vector-borne infectious disease largely spread in Colombia). One of the measures taken was the identification of the dogs affected by canine leishmaniasis. Our project was perfectly matching their objective, therefore in Neiva the health authorities at the departmental level supported the work of our researchers. The field work was done in parallel by the staff of the Public Health authority, which were collecting blood samples from all the dogs from the affected areas for performing the rapid immunochromatographic test against rK39 antigen, and by our researchers. The staff of the Public Health authority from Neiva has also applied euthanasia to the dogs diagnosed with leishmaniasis.

In the Department of Norte de Santander, where the cases of leishmaniasis are only sporadic, the situation was different. The Public Health authority at departmental level didn't get actively involved in our study, but there were rather the representatives of the local health services from the villages where isolated cases of cutaneous canine leishmaniasis had occurred who showed higher interest in our project, and assisted us in identifying possible cases of canine leishmaniasis and accompanied us in our field work. Given the more limited financial resources of the health services acting at the local level, the diagnosis of canine leishmaniasis was realized in the Laboratory of Biomedical Sciences of University of Pamplona by microscopy analysis and PCR.

### 4.3. Population involvement

In Colombia, in conformity with the current legislation regarding zoonotic diseases (Decree 2257 from 1986), in addition to the mandatory notification of a zoonotic disease, article 49 imposes the elimination by the health authorities of the animals that present a zoonotic disease. Therefore, every diagnosed case of canine leishmaniasis needs to be communicated to the health services for taking appropriate measures. This legal provision creates reticence among the population when participating to a study that could lead to the diagnosis of a zoonotic disease in their pet, because of the very close link that is normally created between humans and their pets.

Due of this reason, in the Department of Norte de Santander, where the cases of canine leishmaniasis are only isolated, it happened that some dog owners, aware about our study and the day we were coming for dogs' sampling, decided to take their dogs away from their home and did not show up during all the day. Nevertheless, other people more aware about the gravity of this disease and its consequences, traveled even very long distances from their difficultly reachable living places for bringing to us their dogs for testing and sampling.

**Figure 2:**
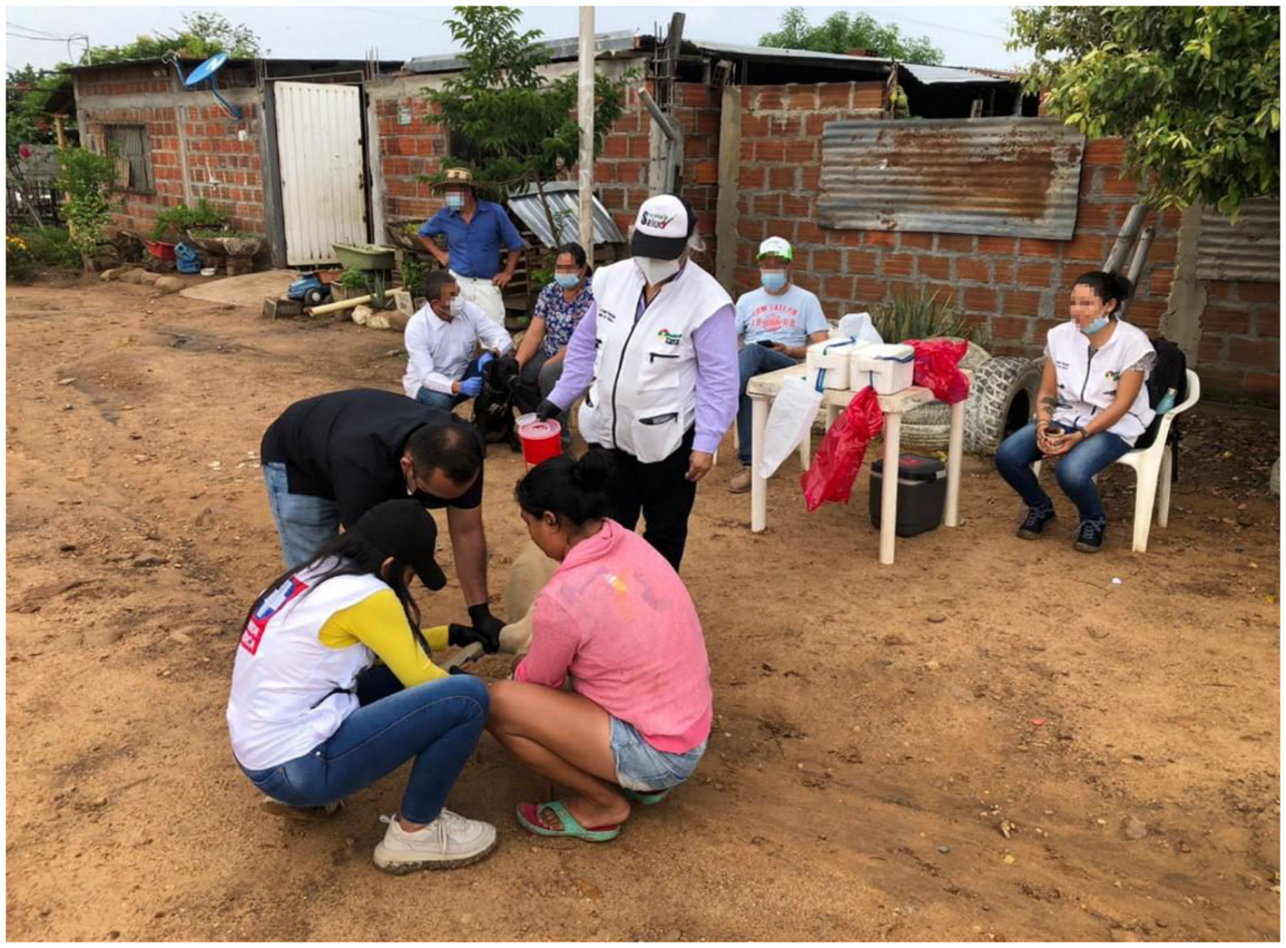
Researchers, public health staff, and local community participating at dogs' sampling.

Instead, in the Department of Huila, the occurrence of such a tragical case as it was the loss of a baby child's life, made all the community aware of the transcendental importance of timely detection of the dogs infected with leishmaniasis. For this reason, the whole community was prone to participate in our study. However, as the sampling was done in the peri-urban area of the city of Neiva, along an illegally built settlement, it was necessary that the representatives of the Public Health Service, who already knew and had contact with the local people, explained them previously the scope of our presence there in order to avoid unpleasant conflictual or other kind of dangerous situations. They became thus very collaborative and participative, and when we arrived there for sampling, the leader of the community announced over the megaphone the local people to bring their dogs at the established meeting point for sampling (Figure 2).

### 4.4. Researchers' challenges

The realization of such a research project that encompassed field work in remote and difficulty accessible communities from a South American country like Colombia implied important challenges for the involved researchers, which ranged from difficult access through roads not properly prepared for car traffic and not lacking of danger, up to people reticence in front of the presence of unknown persons. The great help and assistance received from the staff of the local health services, who accompanied the project researchers on the field and took care of the proper contact with the local people, was essential for the successful realization of this part of the study, which consisted in sampling the volatiles emitted by suspicious dogs for leishmaniasis through breathing and hair. The enthusiasm and no renunciation of the researchers participating in the study were also very important for achieving our objective. Special appreciation in this regard deserves the European researchers from Estonia and Romania, who were not used with the working conditions and customs from a South American country.

Before finishing this perspective point of view, we wish to specially remark the great involvement of the civil servants of the legal authorities from Colombia (Public Health institutions and Mayoralties) and of the local people that, although lacking minimal living conditions, treated us with their highest availability and hospitality. They didn't hesitate any moment to bring us chairs from their houses to sit, or to share with us the scarce food and drinks they had, which was highly appreciated in the torrid tropical days from the Tropics, and in the absence of food and beverage stores. The memories gathered during this joint research project will be never forgotten by the researchers that had the opportunity to live such an amazing experience.

## Data availability statement

The original contributions presented in the study are included in the article/supplementary material, further inquiries can be directed to the corresponding author.

## Ethics statement

The animal study was reviewed and approved by Committee for Ethics and Environmental Impact in Research of University of Pamplona, Colombia (Approval Certificate No. 002 from April 14th, 2021). Written informed consent was obtained from the owners for the participation of their animals in this study.

## Author contributions

All authors listed have made a substantial, direct, and intellectual contribution to the work and approved it for publication.

## Footnotes

^1^Administrative territorial division in Colombia.
